# Review of Small Gauge Vitrectomy: Progress and Innovations

**DOI:** 10.1155/2017/6285869

**Published:** 2017-05-10

**Authors:** Shaheeda Mohamed, Carl Claes, Chi Wai Tsang

**Affiliations:** ^1^Department of Ophthalmology and Visual Sciences, The Chinese University of Hong Kong, Hong Kong Eye Hospital, Kowloon, Hong Kong; ^2^Sint Augustinus Hospital, Wilrijk, Belgium

## Abstract

*Purpose.* To summarise the surgical advances and evolution of small gauge vitrectomy and discuss its principles and application in modern vitreoretinal surgery. The advent of microincisional vitrectomy systems (MIVS) has created a paradigm shift away from twenty-gauge vitrectomy systems, which have been the gold standard in the surgical management of vitreoretinal diseases for over thirty years. Advances in biomedical engineering and surgical techniques have overcome the technical hurdles of shifting to smaller gauge instrumentation and sutureless surgery, improving surgical capabilities and expanding the indications for MIVS.

## 1. Introduction and History of Microincisional Vitrectomy Surgery (MIVS)

Robert Machemer introduced pars plana vitrectomy (PPV) in 1971. Prior to this, Kasner had described vitreous excision removal under an open sky technique, using sponge and scissors [1]. Machemer developed a closed system for vitreous removal with control of intraocular pressure. His vitreous infusion suction cutter (VISC) was 17-gauge (1.42 mm in diameter), multifunctional, and utilized a 2.3 mm scleral incision [2, 3]. O'Malley and Heintz separated the components of vitreous cutting, infusion, and illumination and developed the first three-port 20-gauge(G) vitrectomy system in 1974. Absorbable sutures were used to close the sclerotomies and conjunctiva [4]. Improvements in electric and pneumatic cutters led to 20G three-port vitrectomy becoming the standard technique for vitreoretinal surgery for over thirty years, until the advent of microincisional vitrectomy surgery (MIVS). In 1985, Machemer and Hickingbotham introduced the first 20G trocar/cannula system to be inserted into the sclerotomy, allowing for easier passage of instruments and reduced traction at the vitreous base [5]. In 1990, De Juan developed 25G instrumentation for use in paediatric eyes [6]. Peyman then developed a 23G vitrectomy probe in 1990, primarily intended for vitreous and retinal biopsies [7, 8]. In 2002, Fujii et al. introduced a 25G transconjunctival vitrectomy system using microtrocars and cannulas and popularized the widespread use of small gauge pars plana vitrectomy [9–11]. Eckardt later introduced 23G vitrectomy instrumentation as an alternative to the 25G system [12], and the trend toward yet smaller gauge instruments continued with the development of a 27G sutureless vitrectomy system by Oshima in 2010 [13].

## 2. Advantages and Disadvantages of Small Gauge Vitrectomy

Small gauge vitrectomy, with its smaller instrumentation intended to be transconjunctival, self-sealing, and sutureless, has theoretical advantages including decreased ocular trauma and inflammation, decreased corneal astigmatism, reduced operating times, faster postoperative recovery, increased patient comfort, reduced conjunctival scarring, and conjunctival preservation, especially in patients with prior or pending glaucoma surgery [14–16]. In addition, smaller gauge vitrectomy instruments are better suited to the narrower spaces of paediatric eyes.

However, miniaturization of instruments limits instrument diameter and lumen, with counterproductive effects on instrument flexibility, efficiency, and performance. Initial indications for small gauge vitrectomy were limited to those not requiring extensive vitrectomy, membrane dissection, or phacofragmentation, due to initial issues with limited instrument array and increased flexibility. Advances in wound construction, instrumentation, fluidics, cutter technology, illumination, and wide-angle viewing systems (WAVS) have overcome the handicaps of smaller gauge instrument size and are discussed in detail as follows.

## 3. Trocar/Cannula System

Standard 20G vitrectomy surgery requires conjunctival incisions and sclerotomies of 0.89 mm diameter. Smaller gauge vitrectomy using transconjunctival trocar/cannula systems, have reduced the scleral incision diameter to 0.64 mm for 23G, 0.51 mm for 25G, and 0.4 mm for 27G. The trocar/cannula system theoretically creates less traction on the vitreous base during instrument entry and exit. However, a large retrospective study by Rizzo et al. found similar incidence of retinal detachment after sutureless 23G and 25G vitrectomies and conventional 20G vitrectomy (1.7% versus 1.2%) [17]. The once only placement of the cannulas maintains the alignment between the conjunctiva and sclera and is less traumatic to wound borders than the repeated insertion and withdrawal of instruments through a 20G sclerotomy. It also increases the chances of self-sealing sclerotomy closure and minimizes the risk of suture-related inflammatory reaction, or subsequent atrophy and thinning over the sclerotomy site. Cannulas also allow for interchangeability of instrument and infusion sites, allowing for improved access in certain instances.

However, the cannula sleeve internal diameter limits the radius of curvature of intraocular scissors, resulting in a blunted curve and shorter blades, and renders them less efficient for membrane cutting and dissection than their 20G counterparts, forcing surgeons to use other methods [18]. The cannula sleeve may also slightly affect instrument rotation and flexion during globe manipulation, as well as anterior and peripheral access. Placing the sclerotomies closer to the horizontal meridian reduces the need to rotate instruments significantly for peripheral and superior access and avoids displacement of the infusion as the eye is rotated inferiorly [19].

## 4. Wound Construction

Wound construction in smaller gauge vitrectomy systems is a critical step and affects whether the sclerotomy seals well at the end of surgery. The thickness of the sclera in the area of the pars plana is 0.8 mm. Early incisions were straight (perpendicular to the sclera) in 25G systems, as better self-sealing was expected with the smaller diameter incisions [11]. This was changed to an angled (oblique) scleral incision after studies showed better wound closure and reduced risk of hypotony compared with straight incisions [20–22]. One- and two-step techniques of angled wound construction have been described.

The original two-step technique for 23G vitrectomy, as described by Eckardt, involves displacement of the conjunctiva and stabilization of the eye with a pressure plate, followed by use of a sharp angled MVR blade to create the initial slit opening in the sclera followed by insertion of the blunt trocar, onto which the cannula is mounted [12]. This technique allows more consistent wound creation, but it may sometimes cause difficulty in finding the initial point of trocar insertion. The modern one-step technique involves entry by a sharp trocar with a mounted cannula. Cannulas are quick to insert and easily removed from the trocars without need for a second instrument, but it may be necessary to apply a slightly higher pressure to insert the microcannula at an oblique angle, which can cause problems in eyes with recent corneal or scleral wounds [19].

Additional modifications have been made to the one-step angled technique to improve wound architecture. In general, the longer (more oblique) the intrascleral path, the better the wound apposition. In Zorro's incision, the blade is inserted obliquely at an angle of 10 to 15 degrees and enters the vitreous without straightening [23]. Pollack improved on this by suggesting a biplanar incision, where the trocar is inserted at a 5-degree angle to the sclera until 50% scleral depth, and then raised to a 30-degree angle to the sclera. Trocar entry at 30 degrees produces a longer tunnel length of 1.414 mm, compared with a tunnel length of 1.154 mm produced by trocar entry at 45 degrees. The 30% increase in tunnel length results in more watertight closure [23]. Alternatively, the trajectory may also be made very tangential to the sclera at about 5 degrees and then tilted up to a more perpendicular angle after the cut is made through the sclera in order to avoid impaling the retina. Moreover, older blades created chevron-shaped incisions with a tendency to gape, but newer blades create flat, linear incisions [18].

The course of angled incisions can run perpendicular or parallel to the limbus. Kwok et al. modified the original perpendicular incision by rotating the sclera tunnel by 90 degrees, making it parallel to the limbus [24]. Due to the orientation of scleral fibres around the cornea, scleral incisions made parallel to the limbus offer a theoretical benefit of displacing the scleral fibres rather than cutting them, as in incisions that run perpendicular to the limbus, facilitating more rapid and superior sclerotomy closure [25, 26]. In addition, scleral tunnels that run parallel to the limbus are less likely to encroach on the lens or retina. Microincisional scleral tunnel entry radial to the limbus leaves more room for future sclerotomies than conventional 20G incisions running parallel to limbus, preventing coalescing of wounds in repeat surgeries.

Conjunctival and scleral vessels should be avoided where possible, to reduce postoperative subconjunctival haemorrhage. Conjunctival displacement from the scleral incision has been proposed in order that the two incisions will not be aligned after cannula withdrawal, and the conjunctiva will cover the sclerotomy. It is intended to reduce the risk of postoperative scleral wound contamination. However, Singh et al. demonstrated that conjunctival displacement did not prevent ocular surface fluid from entering sutureless 25G scleral incisions in cadaver eyes [27]. Avoiding conjunctival displacement in eyes with silicone oil fill may also prevent leaking silicone oil from becoming trapped in the subconjunctival and sub-Tenon's space [28].

## 5. Valved Cannula System

Newer valved cannula designs remove the need for plugs and consist of a cap-like silicone membrane mounted onto the cannulas (DORC, Dutch Ophthalmic Research Corporation, Zuidland, the Netherlands), or built into the cannula head (Alcon, Fort Worth, Texas, US). They help maintain a closed system, provide more stable intraocular pressure (IOP) control during instrument exchange, and reduce the amount of infusion. High infusion flow can cause turbulence when working with perfluorocarbon liquids, direct mechanical trauma to the retina, ballooning of the retina if the infusion is directed toward a retina break, or increased dehydration if fluid-air exchange has already been performed. Valved cannulas address the problem of high flow from the infusion through open cannulas during instrument exchange due to IOP compensation features, which can lead to a “fountain effect” at the open cannulas and dislodge plugs, or cause vitreous or retinal incarceration at the sclerotomies [29].

However, valved cannulas can lead to increased friction between the instrument and the valve, and difficult entry for soft or flexible tip instruments, such as the soft tip backflush cannula, or the diamond dusted membrane scraper (DDMS). Entry of such instruments requires straight entry at the centre of the valve aligned with the cannula direction, or a second instrument to act as a glider displacing the valve leaflet [30]. Other alternatives are cutting or removing the soft tip and using newer retractable versions of flexible tip instruments, such as the DDMS. A built-in valved cannula design can also create intraocular pressure buildup during air-silicone oil exchange, and venting extensions that allow air to go through the valves have been introduced to prevent this. For DORC valved cannula systems, the silicone caps can be easily popped off to enable passage of soft-tipped instruments, or to allow for venting.

## 6. Transconjunctival Sutureless 20G Entry

Cannulated and noncannulated transconjunctival sutureless entries for 20G systems have also been developed to allow use of the traditionally more rigid scissors, forceps, and cutters and to allow hybrid 20G/25G or 20G/23G approaches, such as dropped nucleus or intraocular foreign body (IOFB) removal. In 1996, Chen et al. introduced self-sealing sclerotomies using scleral flaps for 20G vitrectomy [31]. Single-step and two-step entry 20G transconjunctival cannulated systems (TCS) are commercially available. Lafeta and Claes described a two-step entry for 20G valved TCS (DORC, Zuidland, the Netherlands) using limbus-parallel 3.5 mm scleral tunnels made at a 10-degree angle to the sclera with a bent stiletto, without conjunctival displacement [32]. None of the eyes required suturing, although it should be noted that 92% of eyes received air tamponade. Only one eye developed hypotony (defined as IOP less than 6 mmHg). Single-step beveled entry nonvalved 20G TCS (Synergetics, O'Fallon, MO) has been reported by Kim et al. and Shah et al. However, 35% to 38% of eyes required suturing [33, 34].

## 7. Cannula Removal and Wound Closure

The self-sealing ability of a sclerotomy wound is affected by wound architecture, scleral tunnel length, scleral elasticity, wound apposition by residual vitreous, surface tension of a gas bubble, and intraocular pressure. To facilitate approximation of the wound edges, the cannulas should be withdrawn in a tangential trajectory. Infusion pressure can be decreased prior to cannula removal to minimize vitreous prolapse [19]. Infusion pressure may be activated to raise internal pressure while concurrent external pressure on the wound facilitates the angled incision tunnel to collapse and close [32]. Some vitreous remnant may also plug the ports to an extent during cannula removal. However, there is no increased rate of retinal detachment attributable to this [17]. Removal of the cannula over a nonhollow probe such as a light pipe has been proposed as a means to decrease vitreous wick incarceration. However, competency of scleral closure may be affected [35]. Partial fluid-air exchange may help reduce wound leak from the sclerotomies until fibrin seals the wounds, due to the increased surface tension of gas compared to fluid [19]. However, better wound construction has obviated its routine use in MIVS. If a wound leak is still detected at the end of surgery, absorbable sutures can be placed, especially in the setting of leaking silicone oil. Leakage from sclerotomies is more likely in highly myopic eyes with low scleral rigidity, in eyes with scarred conjunctiva or sclera from previous surgery, in Marfan's syndrome [36], and in young children [19].

## 8. Instrument Rigidity, Functionality, and Array

Rigidity of instruments is dependent on material, thickness, diameter (gauge), and length [37]. As the trend toward smaller gauge continued, problems with instrument array and tool flexure arose. Initially, 25G vitrectomy was primarily utilized in macular surgeries. As the range of instruments for small gauge systems increased, surgeons applied 25G and 27G systems to cases requiring more extensive peripheral vitrectomy, and flexibility of the smaller cutter was a problem, especially when using the instruments to affect eye rotation for peripheral access and visualization. Hubschman et al. demonstrated that 23G and 25G cutters were less stiff than 20G cutters. Even within the same gauge group, cutter stiffness varied due to differences in internal diameter among 25G and 23G vitrector probes [38]. Paradoxical movements at the tip of thinner forceps can also occur since stress on the shaft near the proximal end of the forceps can cause a reverse movement of the distal end during attempted rotation of the eye. Some surgeons stabilize the smaller gauge instruments with an extra finger close to the sclerotomy to reduce bending. Optimal positioning of the sclerotomies close to the horizontal meridian, avoiding the supraorbital rim and bridge of the nose, wide-angle viewing systems, and scleral depression, all minimize the need for eye rotation and problems related to tool flexure.

Newer generation 25G and 27G cutters, endoilluminators, and laser probes are now stiffer, and newer forceps are shorter to increase stiffness. However, shorter instruments may not be suited for use in highly myopic eyes with long axial lengths. Oshima et al. shortened the 27G cutter from 32 mm to 25 mm, with similar rigidity to the 25G cutter, but were still able to perform core and peripheral vitrectomy in eyes with axial lengths ranging from 22 to 28 mm [13]. Tapered stiffening sleeves have also been developed as another means to increase rigidity of the thinner 27G instruments. Besides shortening, the radius of curvature of curved instruments is also often blunted in order to accommodate passage through the narrower internal diameter of the cannulas. As a result, 25G curved scissors are less efficacious than larger 20G scissors, and dissection of dense membranes may need to be completed by other methods [23].

Due to the improvements in instrument stiffness, instrument array in the smaller gauge systems has expanded accordingly, as well as application to a wider range of surgical indications, including simple and complex retinal detachments, macular surgeries, tractional retinal detachments, and stages 4 and 5 retinopathy of prematurity [39–45]. The 27G vitrectomy platform now has an extensive instrument portfolio including valved trocars, light pipe, cutter, backflush brush, forceps, straight scissors, laser, and diathermy. Phacofragmentomes for removal of dense dropped nuclear fragments have traditionally been limited to 20G, but a 23G fragmentome has recently been introduced (DORC, Zuidland, the Netherlands).

## 9. Fluidics of Vitrectomy

### 9.1. Infusion Flow Rates

Reduction in internal diameter of the infusion cannula in smaller gauge systems increases frictional forces and loss of pressure head and decreases volume flow at the infusion tip entry into the eye, as per Poiseuille's law, which states that flow of an incompressible viscous fluid is proportional to the fourth power of radius of the transmitting tubing and inversely proportional to its length [29]. The volume flow rate decreases by a factor of sixteen when the inner tubing radius is reduced by half. In addition, the volume flow rate is directly proportional to the pressure differential and inversely proportional to the fluid viscosity.

Higher infusion pressures in the range of 40–50 mmHg may be a way to compensate for this and allow higher flow rates in smaller gauge systems, but may affect eyes with compromised ocular perfusion [19]. Infusion fluid can be infused into the eye either by a gravity-fed system or a pressurized system. In gravity-fed systems, infusion pressure, measured in centimetres of water, is equivalent to the bottle height above the eye. In vented gas-forced infusion systems, the infusion bottle itself is pressurized and allows for rapid infusion pressure control via console-controlled venting [29]. In the Constellation system (Alcon, Fort Worth, Texas, US), the infusion is pressurized within the console cassette, which should ideally be placed at eye level. The integrated pressurized infusion has internal, noninvasive sensors that constantly measure flow into the eye through the infusion line and cannula and integrate it through the microprocessor of the computer. The resistance is measured during machine priming. Ohm's law for fluids is analogous to Ohm's law for electricity and states that pressure (gradient) is equal to flow rate multiplied by resistance. Vitrectomy creates a pressure gradient that the machine senses and compensates for by increasing infusion. Infusion pressure can therefore be adjusted according to the sensed flow rate to maintain the desired IOP during surgery, and IOP compensation is accurate to within 2 mmHg [37].

### 9.2. Cutter Flow Rates

Vitreous cutters developed based on the VISC had different drive systems. The electric cutter maintained a constant duty cycle (percentage of time the cutter port is open relative to each cutting cycle) with increased cut rate, but it was heavy and the electric motor in the handpiece led to easy muscle fatigue. The pneumatic cutter was first reported by O'Malley and Heintz in 1975 [4]. Until quite recently, pneumatic cutters employed a single pneumatic pulse from a pneumatic energy source located in the machine to close the cutter guillotine blade and relied on a spring to open it to complete a duty cycle. Pneumatic cutters were smaller and lighter, but as the mechanical properties of the spring remain constant, as the cut rate increased, the inability of the spring to keep up with the pneumatically driven closure increased the time the port is closed, thereby decreasing the duty cycle [46].

Engineering advances led to newer dual pneumatic drive cutters, which replaced the passive spring return phase with a second pneumatic piston that actively pushes the guillotine blade into the open position. This allowed a higher duty cycle at ultrahigh-speed cut rates up to 7500 cuts per minute and allowed surgeons to vary the duty cycle between 50% (50/50), less than 50% (shave mode), or more than 50% (core mode) [18]. The latest twin duty cycle (TDC) cutter design on the Enhancing Visual Acuity (EVA) vitrectomy system (DORC, Zuidland, the Netherlands) has a second port in the internal guillotine blade of the pneumatic cutter. The concept of a double-port cutter was originally patented by Hayafuji more than 20 years ago. With two cutting edges, it cuts both forward and backward, nearly eliminating any port closed time, resulting in a 92% duty cycle independent of cutting speed and allowing cut rates to be doubled to reach 16,000 cuts per minute. With the smaller 27G cutters, increased cutting rate and duty cycle improve cutting efficiency, without unduly increasing tractional forces [47].

Cutter size, speed (cut rate), duty cycle, internal probe diameter, and cutter geometry (including port diameter, distance between the port and tip), all affect its performance. The internal diameter of vitrector probes has decreased from 0.52 mm for 20G, 0.36 to 0.39 mm for 23G, 0.26 to 0.29 mm for 25G, and 0.20 mm for 27G systems. It should be noted that the smaller gauge cutters may show some variability of internal diameters [38]. Larger port diameters, such as in the newer Ultravit 25G+ or 27G+ systems, allow higher flows [29]. While the external diameter of the cutter handpiece has dropped from 0.9 mm for 20G to 0.4 mm for 27G, the port diameter of the 27G cutter still reaches 60% that of the 20G probe. The ports of 23G, 25G, and 27G cutters are also significantly closer to the tip of the probe compared with 20G cutters [18]. Smaller 25G and 27G cutters, with port openings close to the tip, can get extremely close to the retina with smaller sphere of influence on surrounding tissue [48]. This not only enhances safety during vitreous shaving over mobile retina but also can allow the cutter to serve as a dissection tool by enabling access to the very narrow tissue planes during membrane dissection in diabetic tractional detachments [49].

Flow rate through the cutter is influenced by the infusion pressure, aspiration pressure, port diameter, internal diameter of the probe, drive mechanism of the cutter, duty cycle, and viscosity of the aspirated vitreous [50]. Adding to the complex interactions, the vitreous itself is a heterogenous substance that exhibits viscoelastic properties. It is elastic and deformable, and its attachments to the retina require the vitreous to be cut as it is aspirated in order to reduce traction on the retina. The vitreous is 98% water, and the rest is composed of a matrix of collagen fibrils, hyaluronic acid, proteins, and glycoproteins. Since it does not behave as a liquid, other factors such as aspiration pressures, cut rates, and duty cycle govern cutter flow rates in clinical settings [50]. Efficient surgery requires the ability to control outflow through the cutter to achieve high flow, such as during core vitrectomy or induction of a posterior vitreous detachment, and conversely, to enable low flow, such as during peripheral vitreous shaving over a detached retina. In both situations, high cut rates are desirable to reduce pulsatile traction on the retina.

Higher flow rates in smaller gauge cutter systems can be achieved by higher aspiration vacuums in the range of 400–600 mmHg to counter the higher pressure head loss with the smaller vitrector probe diameters. Duty cycles with a longer port open time also result in higher flow rates for vitreous removal. With pneumatic cutters, the duty cycle converges at 50% with increasing cut rate for both open biased and closed biased duty cycles. However, it is important to note that high cut rates reduce the “bite” size and thus the effective viscosity of non-Newtonian fluids such as vitreous. Flow rates and efficiency of vitreous removal can therefore be maintained at high cut rates, and pulsatile traction is minimized [29]. Watanabe et al. have even recently reported removal of dropped nuclear fragments using a 27G TDC cutter and found that the cutter was able to maintain stable suction power to hold the fragments at high cut rates [47].

Vitrectomy systems, such as Constellation (Alcon, Fort Worth, Texas, US), have traditionally used Venturi pumps to create vacuum because the older peristaltic pump designs were constrained by slower rise times than Venturi pumps due to pump inertia and inherently pulsatile flow resulting from rotary compression of the flexible tubing [29]. However, due to advances in peristaltic pump design, some newer vitrectomy machines offer both venturi and peristaltic pumps. With venturi pump systems, the vacuum is set and flow will vary according to viscosity of substances encountered by the cutter. High maximum vacuum can be set for core vitrectomy and low maximum vacuum for peripheral vitreous shaving. In peristaltic systems, it is the flow that is set and vacuum varies to maintain flow with varying viscosity of substances. Similarly, high flow rates can be set for core vitrectomy and low flow rates for vitreous shaving. EVA (Dorc, Zuidland, the Netherlands) has a flow-control technology called VacuFlow Valve Timing Intelligence (VTI) that combines computer-controlled operating pistons and closure valves working in small-flow chambers to allow the surgeon to have adjustable settings for both peristaltic and venturi controls. There is some debate as to whether a peristaltic pump system gives more control during vitreous shaving over a detached retina [51]. However, low flow settings, and automatic adjustment of vacuum and infusion parameters to maintain constant flow, do minimize surge turbulence at the port and traction on surrounding tissue [29]. Furthermore, newer generation MIVS systems offer a dual dynamic drive (3D) vitrectomy mode or a proportional vitrectomy mode. The 3D vitrectomy mode allows for simultaneous linear control of cutting rate and vacuum pressures to produce the resulting flow rate and enhancing efficiency. As the surgeon presses the footpedal, he can change the settings linearly from a preset start point for cutting and the vacuum to a preset endpoint. The proportional vitrectomy mode allows for high fixed cut rates as the vacuum is varied linearly, thereby reducing pulsatile traction and enhancing safety [18].

“Port-based flow limiting,” which describes the flow limitations of cutter gauge size, port size, cut rate, and duty cycle, can be seen as an advantage of smaller gauge systems. A reduced flow rate and high cut rates reduce the average vitreous fibre travel between cuts and therefore limit the traction exerted on the vitreous and retina [29]. A closed biased duty cycle and low flow reduce motion of the detached retina during peripheral vitreous shaving and reduce postocclusion surge after sudden elastic deformation of dense membranes through the cutter port during membrane delamination with the cutter in diabetic tractional detachments [29].

## 10. Illumination

The first light source for vitrectomy originated from an external slit illuminator. In 1976, Peyman introduced endoillumination for 20G vitrectomy using a fibre optic inserted into the vitreous cavity [52]. Coaxial and slit lamp transcorneal illumination from the operating microscope produce scattered light (glare), while endoillumination minimizes light reflections and light scattering from the viewing system lens, cornea, lens, and vitreous [53]. Modes of endoillumination include light pipes, chandelier lights, and illuminated instruments.

Handheld light pipes allow techniques of focal bright illumination, specular illumination where light shone at a critical angle causes an almost transparent surface to glow, highlighting surface irregularities, as well as retroillumination by reflecting the endoilluminator off the surface of the retina, retinal pigment epithelium, choroid, sclera, or off the cutter [53]. Conventional halogen or metal halide light sources initially caused decreased illumination with the smaller gauge light probes. Compared to 20G light pipes, 23G and 25G endoilluminators had reduced light transmission due to reduced surface area of the fibre optic by 40% and 50%, respectively, and therefore required higher power sources. Initially, high arc lamps (xenon and mercury vapour) provided the high power output required for small gauge endoilluminators [53]. Newer light emitting diode (LED) light sources provide up to 40 lumens without degradation of light output, can last more than 10,000 hours, and are therefore particularly suited for smaller gauge endoillumination. Moreover, newer light probes have wider cone angles and allow better peripheral viewing with less probe angulation. Some newer generation light probes offer more than 100 degrees divergence angle, compared to older generation endoilluminators with illumination fields ranging from 50 to 80 degrees. Beveled sheath designs on the tips of some help to minimize glare, while providing wide-angle illumination.

Chandelier light and illuminated instruments were developed to allow bimanual surgery. Illuminated picks provide focal bright light at the surgical dissection site, allowing clearer delineation of the surgical dissection planes. Illuminated lasers, scissors, forceps, and infusions are also available. Chandelier illumination is fixed in the sclera and provides wide-angle diffuse lighting. Eckardt developed 25G “twin light” chandelier illumination in 2003, introduced through two sclerotomies to provide more homogenous lighting and minimize shadows seen with single fibres [54]. Other 25G endoilluminators include the Tornambe Torpedo (Insight instruments, Stuart, FL) and the Awh 25G chandelier (Synergetics Inc., St Charles, MO). Much brighter xenon light sources, such as BrightStar (DORC, Zuidland, the Netherlands), Photon Light Source (Synergetics Inc., St Charles, MO), or integrated into vitrectomy machines such as Constellation (Alcon, Fort Worth, Texas, US), allowed the development of smaller gauge chandeliers. Oshima developed a self-retaining 27G chandelier endoilluminator in 2007 [55], and Eckardt then introduced a 27G twinlight chandelier illumination system [56]. A 30G dual fibre chandelier system in 29G cannulas (Synergetics Inc.,St. Charles, MO) is now also available [57].

While chandelier lights produce superior video image quality, the more distant diffuse fixed illumination may be less helpful in identifying dissection planes at the surgical point of interest and cause glare after fluid-air exchange. Excessive use of diffuse illumination also reduces the ability to see transparent structures such as the internal limiting membrane (ILM), clear epiretinal membranes (ERM), and the vitreous, compared to focal illumination from light pipes [53]. Shadows cast by instruments in the path of the light may impede visualization, and thermal buildup has also been known to occur in the steadily illuminated chandelier [58].

Phototoxicity from high intensity light sources can be reduced by starting with low intensities, lowering intensities when switching from 25G to 20G endoilluminators, shortening exposure times to the macula, and maximizing working distances between the tip of the endoilluminator and the retina [53]. Newer LED light sources, used in LEDStar (DORC, Zuidland, the Netherlands) and integrated in EVA (DORC, Zuidland, the Netherlands), have built-in adjustable yellow filters to minimize phototoxicity.

## 11. Wide-Angle Viewing Systems (WAVS)

In conjunction with developments in MIVS, enhancements in wide-angle viewing systems (WAVS) have improved panoramic viewing of the surgical field and enhanced safety and efficiency. They reduce the need for eye rotation, head repositioning, or scleral indentation and are particularly advantageous when using the smaller gauge cutters. Most WAVS consist of two components: an indirect ophthalmoscope lens placed on or above the cornea and a prismatic stereo reinverter that reinverts the image. WAVS are broadly classified into contact and noncontact viewing systems. Contact WAVS have a fixed field of view depending on the lens dioptric power, whereas the field of view in noncontact systems varies depending on the distance between the ophthalmoscope lens and the cornea [59]. Two contact-based wide-angle lens systems, ClariVit and HRX (Volk Optical Inc), are available. They provide approximately 10 degrees wider field of view than noncontact systems and provide superior image quality as they eliminate corneal aberrations and light reflections by directly placing the lens on the cornea. However, an experienced assistant is needed to hold the lens, and therefore more surgeons prefer to use the noncontact systems. Noncontact systems widely used include BIOM (Binocular Indirect Ophthalmo Microscope; Oculus, Wetzlar, Germany), OFFISS (Optical Fibre Free Intravitreal Surgery System, Topcon Medical Systems, Oakland, NJ), Resight 700 (Carl Zeiss Meditec AG, Jena, Germany), and Peyman-Wessels-Landers vitrectomy lens (Ocular Instruments, Bellevue, WA) [60]. The BIOM system is the most commonly used WAVS, is easily adaptable to most microscopes, and is easily sterilized. The latest version, BIOM 5, offers foot-pedal controlled focusing and automatic image inversion. Newer lenses are wider in diameter, are adapted to compensate for the optical properties of the eye, and have variable back focal length optimized to focus on a curved instead of a flat surface, allowing the concave fundus surface to be in focus for the full extent of the retina from the macula to the periphery with minimal distortion or aberration. However, flat planoconcave contact lenses still have superior axial resolution and lateral resolution over wide-angle systems for macular surgery [53].

## 12. Complications Associated with MIVS

### 12.1. Intraoperative

Rise in intraocular pressure to more than 60 mmHg has been measured during insertion of the trocar cannula complex [60], but newer sharper trocar blade designs have improved ease of entry. Increased intraocular pressure and globe deformation can open recent corneal or scleral wounds, and placing sutures prior to port insertion reduces the risk of wound gaping and hypotony [61]. Displacement of the infusion cannula, during scleral indentation and eye rotation, can lead to serous or haemorrhagic choroidal detachment [62]. It can also occur in eyes with choroidal edema, such as in redetachment surgeries [63]. The dislocated infusion can be quickly moved to one of the other two ports to repressurize the eye. Avoiding excessively long scleral tunnels and placing the infusion closer to the horizontal meridian prevent easy dislodgment by the inferior lid or speculum. Cannula dislodgement can occur when instruments are withdrawn from the eye as a result of increased friction between the instrument and cannula wall, such as when removing forceps or scissors without fully closing the jaws [19]. Sclerotomies situated over areas of scleral thinning, such as in eyes with repeat surgeries, may have reduced friction between the cannula and the sclera and predispose to cannula dislodgement [23]. The dislodged cannula can be mounted onto a trocar and reinserted through the same scleral tunnel, or if it cannot be found, a new sclerotomy can be made. Rarely, breakage and intraocular dislocation of a segment of a cannula tip have also been reported [63]. Use of hybrid 20G/23G or 20G/25G systems, such as during phacofragmentation, can create infusion/outflow mismatch if care is not taken to raise infusion pressures to match egress [64]. Entry site breaks are not common in small gauge vitrectomy [65, 66]. Gentamicin retinal toxicity has also been reported when given subconjunctivally in eyes undergoing small gauge sutureless vitrectomy and should be avoided [67].

### 12.2. Postoperative

Wound sealing of the sclerotomies was the main problem in the development of sutureless small gauge vitrectomy systems. The hypotony is usually transient, but can sometimes be severe, leading to choroidal detachment or haemorrhage, hypotonous maculopathy, or gas escape, and inadequate tamponade [19, 68]. Furthermore, initial reports suggested higher rates of endophthalmitis. This may be due to contamination from conjunctival flora, ingress associated with postoperative hypotony, and vitreous wick effect at unsutured sclerotomies [69]. India ink passage has been demonstrated in eyes with unsutured 25G, 23G (straight or beveled), and 20G sclerotomies, compared to no entry of India ink in eyes with sutured sclerotomies [21, 70]. Kunimoto et al. reported an endophthalmitis incidence of 0.23% for 25G PPV compared to 0.018% for 20G [71], and Scott et al. identified an endophthalmitis incidence of 0.84% for 25G PPV compared to 0.03% for 20G in their cohort studies [69]. This may have been related to variations in sclerotomy construction, as a straight incision was found to have increased risk of endophthalmitis compared with a beveled approach. A systematic review by Govetto et al. did not find an increased risk of endophthalmitis for microincisional vitectomy systems compared to standard 20G vitrectomy [72].

## 13. Summary and Future Directions for MIVS

Significant strides in microincisional vitrectomy system fluidics, instrumentation, illumination, and viewing systems have been made in recent years, and MIVS has all but replaced 20G systems for a wide variety of vitreoretinal surgical indications. Retinal specialists have shifted away from 20G systems to smaller sutureless systems that have reduced operative times, surgical trauma, inflammation, astigmatism, and improved patient comfort, postoperative recovery times, and patient satisfaction. This drives the quest toward even smaller gauge systems, although this is tempered by the engineering challenges, instrument tradeoffs, surgical learning curves, availability, and, importantly, the higher costs. Careful case selection and optimisation of surgical techniques for small gauge systems are important for surgical success.

## References

[1] Kasner D. (1969). Vitrectomy: a new approach to management of vitreous. *Highlights of Ophthalmology*.

[2] Machemer R., Buettner H., Norton E., Parel J. M. (1971). Vitrectomy: a pars plana approach. *Transactions American Academy of Ophthalmology and Otolaryngology*.

[3] Machemer R., Parel J., Norton E. (1972). Vitrectomy: a pars plana approach—technical improvements and further results. *Transactions American Academy of Ophthalmology and Otolaryngology*.

[4] O’Malley C., Heintz R. M. (1975). Vitrectomy with an alternative instrument system. *Annals of Ophthalmology*.

[5] Machemer R., Hickingbotham D. (1985). The three-port microcannular system for closed vitrectomy. *American Journal of Ophthalmology*.

[6] De Juan E., Machemer R., Charles S., Hirose T., Tasman W. S., Trese M. T. (1987). Surgery for stage 5 retinopathy of prematurity. *Archives of Ophthalmology*.

[7] Peyman G. A. (1996). A pneumovitrector for the diagnostic biopsy of the vitreous. *Ophthalmic Surgery and Lasers*.

[8] Peyman G. A. (1990). A miniaturized vitrectomy system for vitreous and retinal biopsy. *Canadian Journal of Ophthalmology*.

[9] Fujii G. Y., De Juan E., Humayun M. S. (2003). Improvements after sheathotomy for branch retinal vein occlusion documented by optical coherence tomography and scanning laser ophthalmoscope. *Ophthalmic Surgery, Lasers & Imaging*.

[10] Fujii G. Y., De Juan E., Humayun M. S. (2002). Initial experience using the transconjunctival sutureless vitrectomy system for vitreoretinal surgery. *Ophthalmology*.

[11] Fuji G. Y., De Juan E., Humayun M. S. (2002). A new 25-gauge instrument system for transconjunctival sutureless vitrectomy surgery. *Ophthalmology*.

[12] Eckardt C. (2005). Transconjunctival sutureless 23-gauge vitrectomy. *Retina*.

[13] Oshima Y., Wakabayashi T., Sato T., Ohji M., Tano Y. (2010). A 27-gauge instrument system for transconjunctival sutureless microincision vitrectomy surgery. *Ophthalmology*.

[14] Chen E. (2007). 25-gauge transconjunctival sutureless vitrectomy. *Current Opinion in Ophthalmology*.

[15] Spirn M. J. (2009). Comparison of 25, 23 and 20-gauge vitrectomy. *Current Opinion in Ophthalmology*.

[16] Okamoto F., Okamoto C., Sakata N. (2007). Changes in corneal topography after 25-gauge transconjunctival sutureless vitrectomy versus after 20-gauge standard vitrectomy. *Ophthalmology*.

[17] Rizzo S., Beltling C., Genovesi-Ebert F., di Bartolo E. (2010). Incidence of retinal detachment after small-incision, sutureless pars plana vitrectomy compared with conventional 20-gauge vitrectomy in macular hole and epiretinal membrane surgery. *Retina*.

[18] Nagpal M., Paranjpe G., Jain P., Videkar R. (2012). Advances in small-gauge vitrectomy. *Taiwan Journal of Ophthalmology*.

[19] Thompson J. T. (2011). Advantages and limitations of small gauge vitrectomy. *Survey of Ophthalmology*.

[20] Hsu J., Chen E., Gupta O., Fineman M. S., Garg S. J., Regillo C. D. (2008). Hypotony after 25-gauge vitrectomy using oblique versus direct cannula insertions in fluid-filled eyes. *Retina*.

[21] Singh R. P., Bando H., OF B., Williams D. R., Kaiser P. K. (2008). Evaluation of wound closure using different incision techniques with 23-gauge and 25-gauge microincision vitrectomy systems. *Retina*.

[22] Taban M., Sharma S., Ventura A., Kaiser P. K. (2009). Evaluation of wound closure in oblique 23-gauge sutureless sclerotomies with visante optical coherence tomography. *American Journal of Ophthalmology*.

[23] Khanduja S., Kakkar A., Majumdar S., Vohra R., Garg S. (2013). Small gauge vitrectomy: recent update. *Oman Journal of Ophthalmology*.

[24] Lopez-Guajardo L., Pareja-Esteban J., Teus-Guezala M. A. (2006). Oblique sclerotomy technique for prevention of incompetent wound closure in transconjunctival 25-gauge vitrectomy. *American Journal of Ophthalmology*.

[25] Kwok A. K. H., Tham C. C. Y., Lam D. S., Li M., Chen J. C. (1999). Modified sutureless sclerotomies in pars plana vitrectomy. *American Journal of Ophthalmology*.

[26] Rizzo S., Genovesi-Ebert F., Vento A., Miniaci S., Cresti F., Palla M. (2007). Modified incision in 25-gauge vitrectomy in the creation of a tunneled airtight sclerotomy: an ultrabiomicroscopic study. *Graefe's Archive for Clinical and Experimental Ophthalmology*.

[27] Singh A., Chen J. A., Stewart J. M. (2008). Ocular surface fluid contamination of sutureless 25-gauge vitrectomy incisions. *Retina*.

[28] Gorovoy I. R., Stewart J. M. (2013). 360 degrees subconjunctival silicone oil after unsutured 23-gauge vitrectomy. *Eye*.

[29] Steel D. H. W., Charles S. (2011). Vitrectomy fluidics. *Ophthalmologica*.

[30] Oellers P., Stinnett S., Mruthyunjaya P., Hahn P. (2016). Small-gauge valved versus nonvalved cannula pars plana vitrectomy for retinal detachment repair. *Retina*.

[31] Chen J. C. (1996). Sutureless pars plana vitrectomy through self-sealing sclerotomies. *Archives of Ophthalmology*.

[32] Lafeta A., Claes C. (2007). 20G transconjunctival sutureless vitrectomy trocar system. *Retina*.

[33] Kim J. E., Shah S. N., Choi D. L., Han D. P., Connor T. B. (2009). Transconjunctival 20-gauge par plana vitrectomy using a single entry cannulated suture-less system. *Retina*.

[34] Shah M., Kapur R., Raja S., Blair M. P. (2011). Transconjunctival 20-gauge vitrectomy outcomes. *Ophthalmic Surgery, Lasers and Imaging*.

[35] Benitez-Herreros J., Lopez-Guajardo L., Camara-Gonzalez C., Silva-Mato A. (2012). Influence of the interposition of a non-hollow probe during cannula extraction on sclerotomy vitreous incarceration in sutureless vitrectomy. *Investigative Ophthalmology & Visual Science*.

[36] Sridhar J., Chang J. S., Aziz H. A., Erickson B. P. (2015). Delayed sclerotomy wound dehiscence after lensectomy and vitrectomy in Marfan syndrome. *Oman Journal of Ophthalmology*.

[37] Nagpal M., Verma A., Goswami S. (2015). Microincision vitrectomy surgery—past, present and future. *European Ophthalmic Review*.

[38] Hubschman J. P., Gupta A., Bourla D. H., Culjat M., Yu F., Schwartz S. D. (2008). 20-23-, and 25-gauge vitreous cutters: performance and characteristics evaluation. *Retina*.

[39] Tsang C. W., Cheung B. T., Lam R. F. (2008). Primary 23-gauge transconjunctival sutureless vitrectomy for rhegmatogenous retinal detachment. *Retina*.

[40] Shah C. P., Ho A. C., Regillo C. D., Fineman M. S., Vander J. F., Brown G. C. (2008). Short-term outcomes of 25-gauge vitrectomy with silicone oil for repair of complicated retinal detachment. *Retina*.

[41] Patelli F., Radice P., Zumbo G., Frisone G., Fasolino G. (2007). 25-gauge macular surgery: results and complications. *Retina*.

[42] Yang S. J., Yoon S. Y., Kim J. G., Yoon Y. H. (2009). Transconjunctival suturelsss vitrectomy for the treatment of vitreretinal complications in patients with diabetes mellitus. *Ophthalmic Surgery, Lasers & Imaging*.

[43] Sen J., Groenewald C., Hiscott P. S., Smith P. A., Damato B. E. (2006). Transretinal choroidal tumor biopsy with a 25-gauge vitrector. *Ophthalmology*.

[44] Gonzales C. R., Boshra J., Schwartz S. D. (2006). 25-gauge pars plicata vitrectomy for stage 4 and 5 retinopathy of prematurity. *Retina*.

[45] Lam D. S., Fan D. S., Mohamed S., Yu C. B., Zhang S. B., Chen W. Q. (2005). 25-gauge trans-conjunctival sutureless vitrectomy system in the surgical management of children with posterior capsular opacification. *Clinical & Experimental Ophthalmology*.

[46] Mimura T., Nakashizuka T., Mor M. (2011). Recent advances and history of vitreous surgery. *Journal of Healthcare Engineering*.

[47] Watanabe A., Tsuzuki A., Arai K., Gekka T., Tsuneoka H. (2016). Treatment of dropped nucleus with a 27 gauge twin duty cycle vitreous cutter. *Case Reports in Ophthalmology*.

[48] Dugel P. U., Zhou J., Abulon D. J. K., Buboltz D. C. (2012). Tissue attraction associated with 20 gauge, 23 gauge, and enhanced 25 gauge dual-pneumatic vitrectomy probes. *Retina*.

[49] Rizzo S., Barca F., Caporossi T., Mariotti C. (2015). Twenty-seven-gauge vitrectomy for various vitreoretinal diseases. *Retina*.

[50] Magalhaes O. J., Chong L. M., DeBoer C. B. (2008). Vitreous dynamics: vitreous flow analysis in 20-, 23-, and 25-gauge cutters. *Retina*.

[51] Adelman R. A., Parnes A. J., Ducournau D., EVRS Retinal Detachment Study Group (2013). Strategy for the management of uncomplicated retinal detachments. EVRS retinal detachment study report 1. *Ophthalmology*.

[52] Peyman G. A. (1976). Improved vitrectomy illumination system. *American Journal of Ophthalmology*.

[53] Charles S. (2008). Illumination and phototoxicity issues in vitreoretinal surgery. *Retina*.

[54] Eckardt C. (2003). Twin lights: a new chandelier illumination for bimanual surgery. *Retina*.

[55] Oshima Y., Awh C. C., Tano Y. (2007). Self-retaining 27-gauge transconjunctival chandelier endoillumination for panoramic viewing during vitreous surgery. *American Journal of Ophthalmology*.

[56] Eckardt C. (2008). 27 gauge twinlight chandelier illumination system for bimanual transconjunctival vitrectomy. *Retina*.

[57] Sakaguchi H., Oshima Y., Nishida K., Awh C. C. (2011). A 29/30-gauge dual-chandelier illumination system for panoramic viewing during microincision vitrectomy surgery. *Retina*.

[58] Shimada H., Nakashizuka H., Hattori T., Mori R., Mizutani Y. (2007). Thermal injury caused by chandelier fiber probe. *American Journal of Ophthalmology*.

[59] Inoue M. (2014). Wide-angle viewing system. *Developments in Ophthalmology*.

[60] Dalma-Weiszhausz J., Gordon-Angelozzi M., Ustariz-Gonzalez O. (2008). Intraocular pressure rise during 25-gauge vitrectomy trocar placement. *Graefe's Archive for Clinical and Experimental Ophthalmology*.

[61] Wong R. W., Kokame G. T., Mahmoud T. H., Mieler W. F., Tornambe P. E., HR M. D. (2010). Complications associated with clear corneal cataract wounds during vitrectomy. *Retina*.

[62] Tarantola R. M., Folk J. C., Shah S. S. (2011). Intraoperative choroidal detachment during 23-gauge vitrectomy. *Retina*.

[63] Chen C. J., Satofuka S., Inoue M., Ishida S., Shinoda K., Tsubota K. (2008). Suprachoroidal hemorrhage caused by breakage of a 25-gauge cannula. *Ophthalmic Surgery, Lasers & Imaging*.

[64] Sallam A., Zambarakji H. J. (2008). Infusion aspiration mismatch during 25-gauge vitrectomy with conversion to 20-gauge vitrector. *Annals of Ophthalmology (Skokie, Ill.)*.

[65] Ehrlich R., Goh Y. W., Ahmad N., Polkinghorne P. (2012). Retinal breaks in small-gauge pars plana vitrectomy. *American Journal of Ophthalmology*.

[66] Recchia F. M., Scott I. U., Brown G. C., Brown M. M., Ho A. C., Ip M. S. (2010). Small-gauge pars plana vitrectomy: a report by the American Academy of Ophthalmology. *Ophthalmoology*.

[67] Cardascia N., Boscia F., Furino C., Sborgia L. (2008). Gentamicin-induced macular infarction in transconjunctival sutureless 25-gauge vitrectomy. *International Ophthalmology*.

[68] Ooto S., Kimura D., Itoi K. (2008). Suprachoroidal fluid as a complication of 23-gauge vitreous surgery. *The British Journal of Ophthalmology*.

[69] Scott I. U., Flynn H. W., Dev S. (2008). Endophthalmitis after 25-gauge and 20-gauge pars plana vitrectomy: incidence and outcomes. *Retina*.

[70] Gupta O. P., Maguire J. I., Eagle R. C., Garg S. J., Gonye G. E. (2009). The competency of pars plana vitrectomy incisions: a comparative histologic and spectrophotometric analysis. *American Journal of Ophthalmology*.

[71] Kunimoto D. Y., Kaiser R. S., Wills Eye Retina Service (2007). Incidence of endophthalmitis after 20- and 25-gauge vitrectomy. *Ophthalmology*.

[72] Govetto A., Virgili G., Menchini F., Lanzetta P., Menchini U. (2013). A systematic review of endophthalmitis after microincisional versus 20-gauge vitrectomy. *Ophthalmology*.

